# Transcription factor c-Rel is indispensable for generation of thymic but not of peripheral Foxp3^+^ regulatory T cells

**DOI:** 10.18632/oncotarget.17079

**Published:** 2017-04-13

**Authors:** Maik Luu, Elena Jenike, Niyati Vachharajani, Alexander Visekruna

**Affiliations:** ^1^ Institute for Medical Microbiology and Hygiene, Philipps University of Marburg, Marburg, Germany

**Keywords:** regulatory T cells, NF-κB, inflammation

## Abstract

The transcription factor c-Rel has been shown to be crucial for development of regulatory T cells (Tregs). Recent studies have reported that the expression of transcription factor Helios in Foxp3^+^ Tregs correlates with thymic origin of these cells (tTregs). Notably, we found that only the Helios^+^Foxp3^+^ Treg cell population was substantially reduced in c-Rel deficient mice. In contrast to a defective tTreg development, we observed an expansion of mucosal Tregs during the induction of acute colitis in *rel^−/−^* mice. Furthermore, we found a preferential accumulation of Helios^−^Foxp3^+^ Tregs in aged c-Rel deficient mice. This unexpected finding, together with the observation that naïve CD4^+^ T cells convert into Tregs *in vitro* in the absence of c-Rel and presence of IL-2, provide an evidence that extra-thymic generation of induced and peripheral Tregs (iTregs and pTregs) is independent of c-Rel. Moreover, the treatment with IL-2/anti-IL-2 mAb (JES6-1) resulted in a widespread increase of Helios^+^Foxp3^+^ Tregs in both wild-type (WT) and *rel^−/−^* mice. These data suggest that exogenous IL-2 administration compensates for defective IL-2 production and reduced tTreg numbers in c-Rel deficient mice. Our findings reveal that c-Rel is essential for the generation of tTregs but not for that of pTregs and iTregs.

## INTRODUCTION

Foxp3^+^CD4^+^ regulatory T cells (Tregs) play a crucial role in the maintenance of immune homeostasis and prevention of excessive immune responses [1–3]. Tregs comprise two subsets with two distinct origins and functions: thymus-derived Tregs (tTregs) and peripherally-induced Tregs (pTregs) that are generated from naïve CD4^+^ T cells by numbers of triggers in the peripheral organs such as gut, skin and lung [4]. Additionally, in the presence of IL-2 and TGF-β1, the conversion from CD25^−^ Foxp3^−^ CD4^+^ T cells into induced Foxp3^+^ Tregs (iTregs) can be achieved in *in vitro* T cell cultures [5]. However, these Tregs are less stable and can lose their Foxp3 expression and suppressive activity after the transfer into mice. Previously, it was shown that DNA methylation status at the *Foxp3* locus is crucial for maintaining a stable Treg lineage. The instable Foxp3 expression by *in vitro* generated iTregs is associated with decreased demethylation of the Treg-specific demethylated region (TSDR) at the *Foxp3* locus, which is highly demethylated within tTreg and pTreg subsets [6–8]. Interestingly, mice with a deletion in TGF-β1 or animals lacking specifically Smad2 and Smad3 in T cells have been show to exhibit normal development of tTregs but have significantly decreased numbers of pTregs [9, 10], suggesting that TGF-β signalling pathway is essential for the maintenance of Foxp3 expression and function of Tregs in peripheral organs. Although the presence of TGF-β1 in the gut is essential for optimal pTreg development, recent findings suggest that some exogenous factors such as gut microbiota-derived short chain fatty acids (SCFAs) facilitate expansion of colonic pTregs by enhancing the acetylation of histone H3 at the F*oxp3* locus [11, 12]. On the other hand, the activation of classical NF-κB signalling pathway has been shown to be crucial for the development of Tregs in the thymus [13–15]. Especially, the Treg cell-intrinsic expression of c-Rel is essential for the thymic induction of Foxp3 [16–19]. Similarly, the atypical inhibitor of NF-κB IκB_NS_ is also involved in the development of Tregs by regulating the transition of thymic immature Treg precursors into mature Tregs [20]. Furthermore, mice lacking proteins involved in NF-κB activation such as PKCΘ, Bcl10, CARMA1 and MALT1 display impaired Treg cell development [14, 21]. These data revealed that NF-κB, although originally described as a pro-inflammatory factor, is one of key regulators of development of tTregs with anti-inflammatory properties. However, c-Rel deficient mice still exhibit moderate Foxp3^+^ Treg cell frequencies in the peripheral organs prompting us to investigate the origin of these cells.

The selective *in vivo* expansion of Tregs has the potential to treat autoimmune diseases. Recently, it has been shown that the treatment of mice with IL-2/anti-IL-2 complex, generated by using JES6-1 antibody, increases the number of Tregs and protects from asthma, experimental autoimmune encephalomyelitis (EAE) and type 1 diabetes [22–24]. These results highlight the central role for IL-2 and Tregs in efficient treatment of autoimmune diseases. The members of Ikaros family of transcription factors, Helios and Eos appear to be specifically expressed in Tregs [25, 26]. A recent study has described Eos as an important factor that directly interacts with Foxp3 and induces chromatin modifications resulting in specific gene silencing in Tregs [26]. Helios has been found to be preferentially expressed in Tregs of thymic origin, thus potentially acting as a crucial marker for the discrimination between tTregs and pTregs. Its expression in T cells does not require the activity of Foxp3, as Helios has also been detected in the subsets of DN2-DN4 thymocytes [25].

Here, we show that c-Rel deficient mice have significantly less Helios^+^Foxp3^+^ but not Helios^−^Foxp3^+^ Tregs. Interestingly, only Helios^+^Foxp3^+^ Tregs were specifically enriched after treatment of WT and even *rel^−/−^*mice with IL-2/anti-IL-2 mAb. Surprisingly, we found a strong accumulation of pTregs in aged *rel^−/−^* mice. Furthermore, the expansion of mucosal Tregs during acute colitis as well as normal generation of iTregs in the absence of c-Rel indicate that this transcription factor is essential for the development of tTregs but not for that of pTregs and iTregs.

## RESULTS

### *In vivo* administration of the IL-2/JES6-1 complex induces expansion of Tregs in c-Rel deficient mice

**Figure 1:**
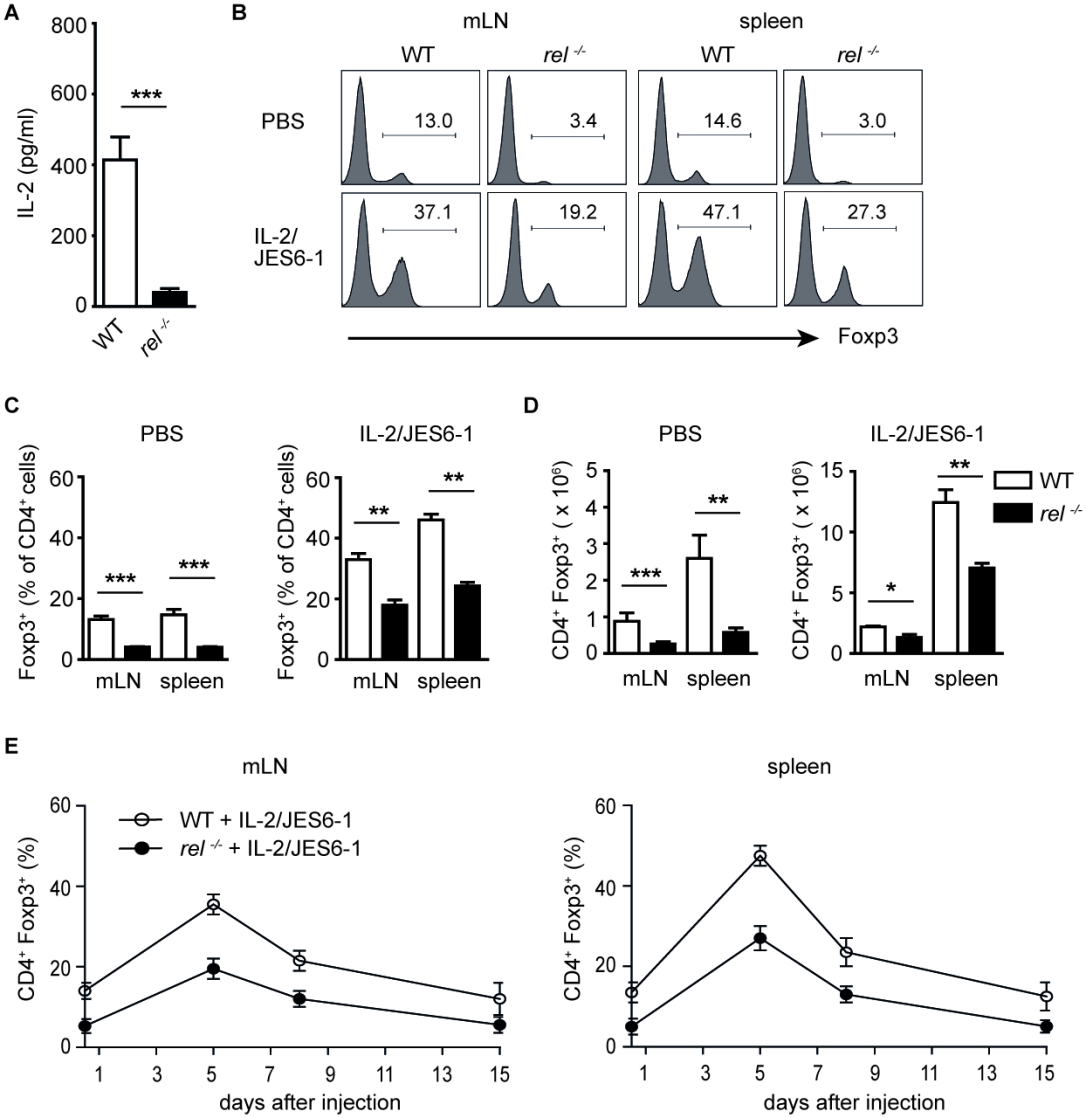
*In vivo* expansion of Foxp3+ Tregs with IL-2/JES6-1 complex in rel−/− mice **(A)** CD4^+^ T cells purified from wild-type (WT) and *rel^−/−^* mice were stimulated with anti-CD3 plus anti-CD28 for 24h. IL-2 secretion was measured by ELISA. Data are means ± SEM; ***P<0.001. **(B)** WT and *rel^−/−^* mice were treated i.p. daily for three days (days 0, 1, 2) with IL-2/JES6-1. The expansion of Tregs was analyzed on day 5 by the intracellular staining of Foxp3 in mLN and spleen. The histograms are gated on CD4^+^ T cells. Data are representative of two independent experiments. **(C)** and **(D)** The percentage (C) and total cell number (D) of Tregs in mLN and spleen on day 5 after treatment of mice with IL-2/JES6-1 (n = 6 mice per group). Results are means ± SEM; *P<0.05, **P<0.01, ***P<0.001. **(E)** A kinetic analysis of Treg expansion after IL-2/JES6-1 treatment of WT and *rel^−/−^* mice. Foxp3 frequencies among CD4^+^ T cells on days 0, 5, 8 and 15 following three injections of IL-2/ JES6-1 are shown (n = 6 per group). Error bars represent SEM.

IL-2 plays an essential role for the thymic development of Tregs and the maintenance of Treg homeostasis in the peripheral tissues [27]. Defective IL-2 production in c-Rel deficient mice was reported previously [28]. Here, we confirm that CD4^+^ T cells isolated from *rel^−/−^* mice and stimulated for 24 h with anti-CD3/anti-CD28 antibodies secrete significantly less IL-2 than their wild-type (WT) counterparts (Figure 1A). Recent studies have shown that Tregs selectively expand after treatment of mice with IL-2 and anti-IL-2 mAb JES6-1 [22, 23]. To analyse whether exogenous *in vivo* supply of IL-2 can reverse Treg-intrinsic requirements for c-Rel, we administered IL-2/JES6-1 complex in WT and *rel^−/−^* animals. Consistent with earlier reports, we found that biological activity of IL-2 is greatly enhanced by association with JES6-1 mAb. When compared to the PBS-treated WT mice, there was a significant increase of Tregs in spleen and mesenteric lymph nodes (mLN) after treatment with IL-2/JES6-1. The proportion of Foxp3^+^ Tregs within CD4^+^ T cell population rose to 30-50 % in these organs as compared to basic level of 10-15 % in PBS-treated animals. Remarkably, the injection of IL-2/JES6-1 also resulted in a strong expansion of Tregs in *rel^−/−^* mice (Figure 1B and 1C). The substantial increase in frequencies of Tregs was also reflected in increased total Treg cell numbers in examined organs (Figure 1D). Kinetic studies demonstrated that three injections of the IL-2/JES6-1 complex resulted in a brief expansion of Tregs which peaked on day 5 and gradually declined on days 8 and 15. This finding refers to both WT and *rel^−/−^* mice, although the Treg expansion in c-Rel deficient mice did not reach the percentage and total cell numbers of WT Tregs (Figure 1E). In conclusion, we found that the short-course treatment of mice with IL-2/JES6-1 led to the substantial expansion of Tregs not only in WT but also in *rel^−/−^* mice, which normally exhibit reduced Treg frequencies and numbers. Taken together, our results demonstrate that signals derived by exogenous IL-2 can partially compensate for defects in c-Rel deficient Tregs.

### Preferential expansion of Helios^+^Foxp3^+^ Tregs after IL-2/JES6-1 treatment

**Figure 2:**
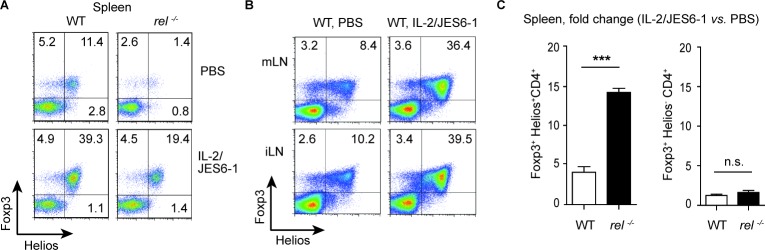
Rapid expansion of Helios+Foxp3+ Tregs after IL-2/JES6-1 treatment **(A)** The effect of the IL-2/JES6-1 complex on expansion of splenic Helios^+^Foxp3^+^ and Helios^−^Foxp3^+^ Tregs was analyzed on day 5 after treatment. Data are representative of three independent experiments. **(B)** IL-2/JES6-1-mediated expansion of Helios^+^Foxp3^+^ Tregs in mLN and inguinal (i) LN on day 5 after treatment of WT mice. A representative of three similar experiments is shown. The dot plots (A and B) are gated on CD4^+^ T cells. **(C)** Data from (A) are shown as a fold change for IL-2/JES6-1-expanded splenic Tregs as compared with Tregs from PBS-treated mice. Results are means ± SEM; n.s., not significant, ***P<0.001.

Previously, it was shown that administration of the IL-2/JES6-1 stabilized the Foxp3 expression and expanded the numbers of transferred iTregs [29]. Although IL-2 administration has been extensively used to boost Treg cell numbers *in vivo*, it is still unknown whether IL-2 specifically expands tTregs or pTregs in the absence of antigen challenge. To investigate if both Treg subsets, tTregs and pTregs, can be expanded by exogenous IL-2, the percentage of Helios^+^Foxp3^+^ and Helios^−^Foxp3^+^ cells was compared among CD4^+^ T cells in lymphoid tissues of WT and *rel^−/−^* mice after IL-2/JES6-1 administration. Surprisingly, the treatment with IL-2/JES6-1 induced strong tTreg but not pTreg cell expansion in spleen, mLN and inguinal (i) LN at day 5 in WT mice (Figure 2A and 2B). Remarkably, this effect was even more pronounced in c-Rel deficient mice. In fact, in the spleen of IL-2/JES6-1-treated *rel^−/−^* mice, approximately a 15-fold increase in the frequency of Helios^+^Foxp3^+^ Tregs was found as compared with naïve animals (Figure 2A and 2C). Furthermore, the significant reduction of Helios^+^Foxp3^+^ but not of Helios^−^Foxp3^+^ Treg population was observed in naïve *rel^−/−^* mice, confirming that c-Rel is indeed a crucial factor required for thymic differentiation of Tregs (Figure 2A).

The unexpected finding that only Helios^+^Foxp3^+^ Tregs robustly proliferate and respond to the supplementation with exogenous IL-2 prompted us to investigate the possible reason for their predominant expansion. IL-2 complexed to JES6-1 has been described to preferentially activate cells that express CD25 and co-injection of CD25-depleting PC61 mAb was able to block Treg cell expansion [23]. Firstly, the treatment of WT mice with CD25-depleting Ab (two i.p. injections of PC61 mAb) targeted both Treg subsets as Foxp3 expression was substantially reduced in Helios^+^ and Helios^−^ Treg populations as compared to control mice (Supplementary Figure 1A). However, when we compared the expression of CD25 for each Treg population, we found that the CD25 expression levels were higher in Helios^+^ Treg than in Helios^−^ Treg population, which suggests that Helios^+^ Treg subset might have a proliferative advantage by responding rapidly to lower IL-2 concentrations (Supplementary Figure 1B). These data are in agreement with a very recent study that shows the preferential expansion of human Helios^+^ Tregs after treatment of patients with low dose IL-2 [30].

### c-Rel is dispensable for the expansion of mucosal Tregs in acute colitis

**Figure 3:**
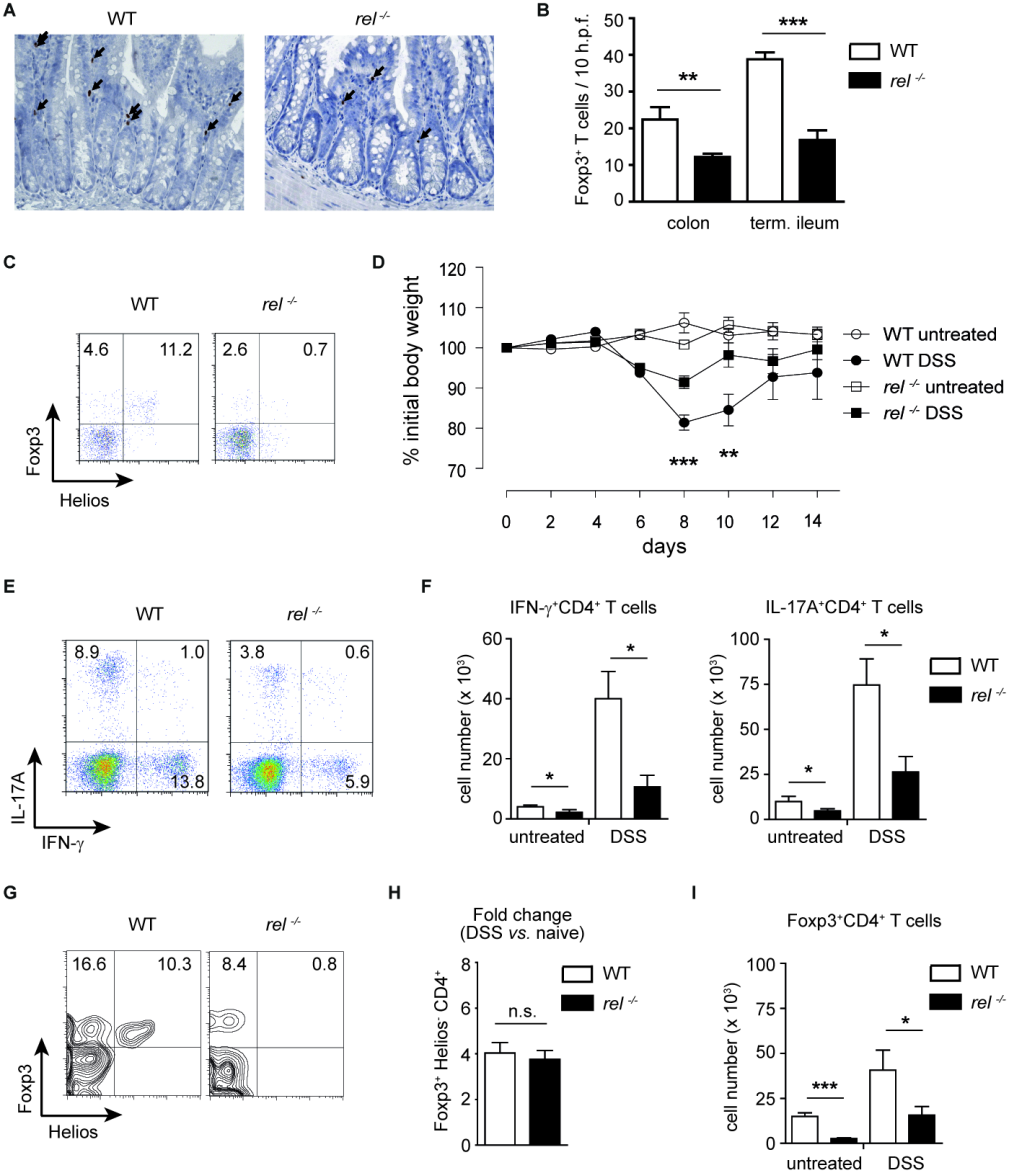
Expansion of mucosal Tregs in rel−/− mice after induction of colitis **(A)** and **(B)** Colons and small intestines from WT and *rel^−/−^* mice were stained for Foxp3 (indicated by arrows) using immunohistochemistry analysis (magnification: 200 x). Data are shown as a number of Foxp3^+^ cells/ 10 h.p.f. (high power fields). Number of animals: 4-5 mice per group. Data are means ± SEM; **P<0.01, ***P<0.001. **(C)** Frequencies of Helios^+^Foxp3^+^ and Helios^−^Foxp3^+^ Treg cells in the colon of naïve WT and *rel^−/−^* mice. A representative of three similar experiments is shown. **(D)** Colitis was induced in WT and *rel^−/−^* mice by orally administering 3 % DSS for 5 days and the change of initial weight was monitored over a period of 14 days. Data represent mean ± SEM (n = 8 mice per group). **P<0.01, ***P<0.001. **(E)** and **(F)** Frequency (E) and total cell number (F) of colonic IFN-γ^+^ and IL-17A^+^ CD4^+^ T cells in untreated and DSS-treated WT and *rel^−/−^* mice, measured by FACS analysis on day 10 after colitis induction. Data in (F) are displayed as means ± SEM; *P<0.05. **(G)** and **(H)** The percentage of Helios^−^Foxp3^+^ Tregs within colonic CD4^+^ T cells after colitis induction by 3 % DSS. Results in (H) are displayed as fold change as compared to naïve mice. n.s., not significant. **(I)** Absolute cell numbers of colonic Tregs in untreated and DSS-treated WT and *rel^−/−^* mice. Results are shown as means ± SEM; *P<0.05, ***P<0.001.

We next performed a histological and flow cytometry analysis of Foxp3 expression in the small intestine and colon of WT and *rel^−/−^* mice. As expected, intestinal Tregs were reduced in c-Rel deficient animals (Figure 3A-3C). Germ-free (GF) mice have also reduced numbers of colonic Tregs that selectively expand after colonization with commensal bacteria [31, 32]. Here, we show that the percentages of colonic Helios^−^Foxp3^+^CD4^+^ cells were almost 5 times lower in GF than in conventional mice, while the percentage of Helios^+^Foxp3^+^ subset was unchanged (Supplementary Figure 2A and 2B). Therefore, it is tempting to speculate that, in accordance with the original report [25], the most Helios^−^Foxp3^+^ Tregs are indeed generated in the peripheral tissues.

To analyse the impact of c-Rel on the expansion of mucosal pTregs, we induced acute colitis in WT and *rel^−/−^* animals and tested whether the accumulation of pTregs at gut mucosal sites is dependent on this transcription factor. Following oral administration of dextran sodium sulfate (DSS), the measurement of the loss of initial body weight and colon length revealed a milder disease course in *rel^−/−^* mice as compared to WT animals (Figure 3D, and Supplementary Figure 3A). In our previous study, we found no reduction in expression of effector cytokines by *in vitro* generated Th17 and Th1 cells [18]. In contrast to these data, on day 10 after colitis induction, a decreased production of IL-17A and IFN-γ by colonic CD4^+^ T cells was detected in *rel^−/−^* mice in comparison to WT animals (Figure 3E and 3F). This discrepancy could be attributed to reduced colonic secretion of proinflammatory cytokines such as IL-12, IL-23, IL-6 and IL-1β (which are known to considerably impact on T cell differentiation) in DSS-treated c-Rel deficient mice (Supplementary Figure 3B and 3C). Notably, the FACS analysis revealed a dispensable role for c-Rel for the expansion of colonic Helios^−^Foxp3^+^ pTregs, as in both, DSS-treated WT and *rel^−/−^* mice, the frequency of these cells increased 4-fold as compared to naïve mice (Figure 3C, 3G and 3H). Similarly, the absolute number of colonic Tregs increased during the progression of colitis in both WT and *rel^−/−^* animals (Figure 3I).

### Selective accumulation of Helios^−^Foxp3^+^ Tregs in aged c-Rel deficient mice

**Figure 4:**
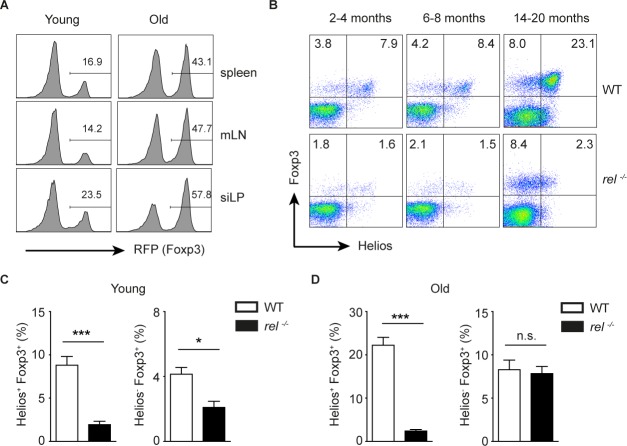
Preferential accumulation of Helios-Foxp3+ Tregs in aged rel−/− mice **(A)** The frequency of RFP^+^ cells within CD4^+^ T cells in spleen, mLN and lamina propria of the small intestine (siLP) in naïve young (8-14 weeks) and old (14-20 months) Foxp3-RFP reporter mice. The cells are gated on CD4^+^ gate. A representative of three experiments is shown. **(B-D)** Young (2-4 months), middle-aged (6-8 moths) and old (14-20 months) naive WT and *rel^−/−^* mice were analyzed for their Helios and Foxp3 expression within splenic CD4^+^ T cells. A representative of two experiments is shown within CD4 gate (B). Data (C and D) are frequencies of indicated cells within CD4^+^ T cell population and are displayed as means ± SEM; n.s., not significant, *P<0.05, ***P<0.001.

During the aging, the immune system undergoes several changes which are not beneficial for the host, including age-related Treg accumulation and suppressed immune phenotype [33]. A recent study has demonstrated that, in addition to the expansion of Helios^−^Foxp3^+^ pTregs, also tTregs with high expression of Helios and Neuropilin 1 (Nrp1) accumulate in aged mice. Furthermore, the aged Tregs were shown to be functional and had a broad V_β_ repertoire [34]. In accordance with previous data, we found increased proportion of Tregs in all investigated tissues in *Foxp3-RFP* reporter mice (Figure 4A). Moreover, we confirmed that the frequency of both Helios^−^Foxp3^+^ and Helios^+^Foxp3^+^ Tregs was increased in aged WT mice as compared to young animals (Figure 4B). Surprisingly, by analysing the Treg subsets in young and old c-Rel deficient mice, we found that in the absence of this transcription factor, the aged Tregs were enriched only in Helios^−^ population (Figure 4B-4D). Taken together, the surprising observation of a substantial increase of pTregs in c-Rel deficient aged mice implies that, in contrary to its crucial role in thymic Treg development, the transcription factor c-Rel is not required for the accumulation of pTregs.

### c-Rel is not required for the differentiation of iTregs in the presence of exogenous IL-2

**Figure 5:**
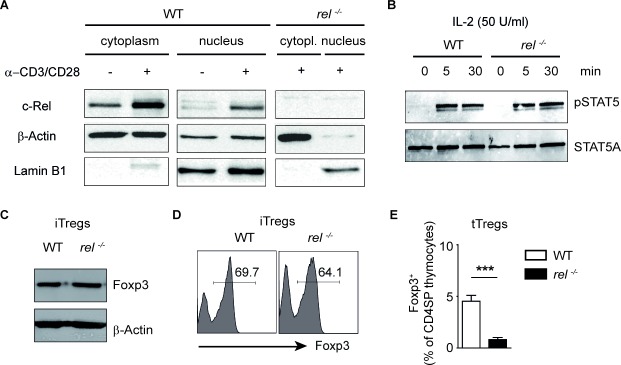
Normal *in vitro* generation of Foxp3+ Tregs in the absence of c-Rel **(A)** Western blot analysis of c-Rel expression in cytoplasmic and nuclear extracts of WT and *rel^−/−^* CD4^+^ T cells after activation with plate-bound anti-CD3 and soluble anti-CD28 antibodies for 24 hours. Lamin B1 and β-Actin served as loading controls. **(B)** Western blot analysis of naïve CD4^+^ T cells purified from spleens of WT and *rel^−/−^* mice and stimulated with IL-2 (50 U/ml) for indicated time points. Phosphorylation of STAT5 was analyzed by using anti-phospho-STAT5 and anti-STAT5A antibodies. Three similar experiments were performed. **(C)** Immunoblot analysis for Foxp3 and β-Actin was performed on day three after culturing the purified naïve CD4^+^ T cells under optimal Treg-inducing conditions (2 ng/ml TGF-β1 and 50 U/ml IL-2). A representative of two experiments is shown. **(D)** FACS Analysis showing the frequency of Foxp3^+^ cells on day 3 of the cell culture. Naïve CD4^+^ T cells were treated as described in (C). **(E)** The percentage of Foxp3^+^ Tregs within CD4^+^CD8^−^ thymocytes (CD4 single positive, CD4SP) in WT and *rel^−/−^* mice. Results are means ± SEM of three independent experiments; ***P<0.001.

It remains obscure whether the observed defective cell numbers of Tregs in peripheral organs of *rel^−/−^* mice are only due to the lack of the thymic activity of c-Rel. We here show that the stimulation of CD3 and co-stimulatory molecule CD28 with anti-CD3/anti-CD28 for 24 hours is sufficient to increase c-Rel expression and translocate this transcription factor into the nucleus of activated CD4^+^ T cells (Figure 5A). To investigate the impact of c-Rel on the generation of iTregs, we isolated naïve CD4^+^ T cells from spleen and LN of WT and c-Rel deficient mice and cultured them in the presence of TGF-β1 and IL-2. Activation of STAT5 upon binding of IL-2 is necessary for subsequent proliferation and survival of Tregs (23). We thus wondered whether this signalling pathway is impaired in T cells lacking c-Rel. In the western blot analysis, a normal phosphorylation of STAT5 was observed in both, WT and c-Rel deficient CD4^+^ T cells treated with IL-2. Thus, although a partial defect in CD25 expression was previously described for *rel^−/−^* T cells [28], CD4^+^ T cells from *rel^−/−^* mice are able to sense the exogenous IL-2 and to response to it by activating STAT5 signalling pathway (Figure 5B and Supplementary Figure 4). Moreover, we were not able to find any defective expression of Foxp3 in T cell cultures lacking c-Rel as compared to WT cells under optimal iTreg-inducing conditions (Figure 5C and 5D). On the other hand, we confirm a defective Treg development in *rel^−/−^* thymocytes (Figure 5E), which was previously observed by several groups [16–18]. Together, the transcription factor c-Rel, although essential for tTreg generation, is dispensable for *in vitro* conversion of naïve CD4^+^ T cells into Foxp3^+^ iTregs in the presence of exogenous IL-2.

## DISCUSSION

The molecular basis of Treg development is characterized by epigenetic regulations within the *Foxp3* locus [35–37]. Investigation of proximal regions at the *Foxp3* locus revealed the existence of several important regulatory elements termed conserved non-coding sequences 1-3 (CNS 1-3) that play a decisive role in the control of *Foxp3* gene expression [19, 38]. Novel findings suggest that c-Rel acts as “pioneer” transcription factor during the Treg development in thymus by binding at the CNS3 region of *Foxp3* locus [14, 19]. Additionally, the expression of IL-2, a cytokine important for Treg homeostasis, is under direct transcriptional control of c-Rel [28, 39, 40]. Steady-state IL-2 levels are indispensable for survival of Foxp3^+^ Tregs [41–45]. Interestingly, we here show that defective Foxp3 expression and Tregs numbers in *rel^−/−^* mice can be partially compensated by adding exogenous IL-2. Two recent clinical studies demonstrate that the administering IL-2 at low doses provides a therapeutic window to promote self-tolerance and suppress adverse immune responses in humans. Both reports suggest that beneficial effects are mediated by boosting Treg cell numbers [46, 47]. By treating mice with IL-2/JES6-1 complexes, we were able to show that the Helios expression in Tregs is linked to the selective, IL-2-mediated enrichment of these cells. Moreover, the increase of Helios^+^Foxp3^+^ Tregs was even more pronounced in c-Rel deficient mice when compared to the naïve animals.

The expression of transcription factor Helios in Tregs has been proposed to be independent from signals required for induction of Foxp3 [48]. Although defining the origin of functional Treg subsets is difficult, Helios has been suggested to be a suitable marker for thymus-derived tTregs [4]. Recent two studies have demonstrated that also Neuropilin 1 (Nrp1) is specifically expressed on tTregs but not on pTregs [49, 50]. While these reports showed a strong correlation between Nrp1 and Helios expression exclusively on tTregs, other researchers challenged this concept demonstrating that Helios might be involved in various cellular processes in T cells such as activation, proliferation, apoptosis of autoreactive thymocytes, Th2 development as well as T cell anergy and exhaustion [51–55].

**Figure 6:**
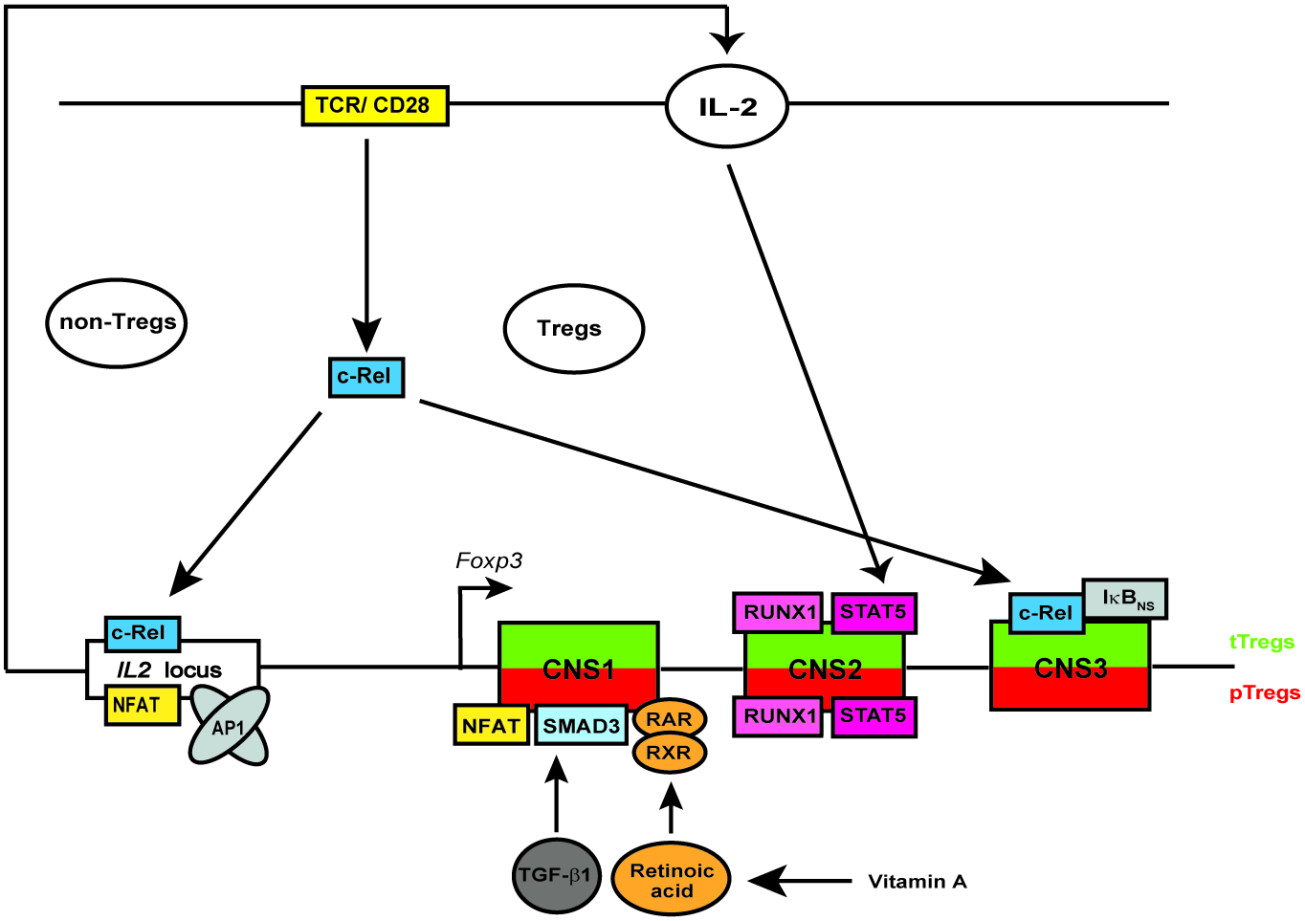
Schematic overview of the proposed mechanism of c-Rel-mediated regulation of Tregs c-Rel regulates the Treg development and homeostasis by binding to the *IL2* locus in non-Tregs and to the *Foxp3* locus in developing thymic Tregs, respectively. c-Rel binds together with IκB_NS_ to conserved non-coding sequence 3 (CNS3) of *Foxp3* locus. Several other transcription factors such as STAT5, RUNX1 as well as NFAT, SMAD3, RAR and RXR can bind to CNS2 and CNS1, respectively. Tregs in the peripheral organs and in *in vitro* CD4^+^ T cell cultures develop independently of c-Rel. Abbreviations: TCR, T cell receptor; TGF, transforming growth factor; STAT, signal transducer and activator of transcription; NFAT, nuclear factor of activated T-cells; AP1, activator protein 1; RUNX1, runt-related transcription factor 1; RAR, retinoic acid receptor; RXR, retinoid X receptor.

In this study, we observed more prominent reduction of Helios^+^Foxp3^+^ than of Helios^−^Foxp3^+^ Treg population in c-Rel deficient mice. The transcription factor c-Rel has been shown to regulate multiple steps in the thymic development of Foxp3-expressing CD4^+^ Tregs [56]. We and others have recently shown that mice lacking c-Rel possess only 15-25 % of normal Treg cell numbers and frequencies in the thymus and peripheral lymphoid organs [13, 15–18]. Interestingly, the remaining c-Rel deficient Tregs have normal immune suppressive properties *in vivo* [16]. In view of evidence of preferential accumulation of Helios^−^Foxp3^+^ pTregs in aged *rel^−/−^* mice and normal iTreg generation and expansion of mucosal Tregs in the absence of c-Rel, we suggest the following model of c-Rel-dependent and -independent regulation of Tregs: in the thymus, the development of Tregs requires the triggering of the T cell receptor (TCR), whereby the transcription factor c-Rel links the TCR-mediated signals to the induction of *Foxp3* expression. c-Rel might bind to CNS3 in cooperation with other NF-κB-associated molecules such as IκB_NS_ and p50 [20]. Additionally, the IL-2 production by non-Treg cells, which is needed for survival and proliferation of both tTregs and pTregs, is also transcriptionally regulated by c-Rel [28, 40]. IL-2 stimulation of Tregs activates STAT5 that binds together with RUNX1 to CNS2 (also known as TSDR) regulatory element of *Foxp3* locus, which is an important step for stable *Foxp3* expression and maintenance of Treg subsets. On the other hand, in pTregs and iTregs, the c-Rel-independent binding of SMAD3 and NFAT (which also binds together with AP1 to the *IL2* promoter and controls *IL2* gene expression, [57]) to CNS1 is sufficient to generate Tregs in an extra-thymic manner (Figure 6). Additionally, retinoic acid-activated heterodimers consisting of RAR and RXR are also able to bind to CNS1 to induce differentiation of pTregs [58–60].

## MATERIALS AND METHODS

### Mice

Conventional C57BL/6 mice were purchased from Charles River Laboratories (Sulzfeld, Germany) and kept under specific pathogen-free (SPF) conditions. Germ-free (GF) mice on C57BL/6 background, *Foxp3-RFP* reporter mice and *rel^−/−^* mice were bred at the animal facility of the Biomedical Research Center (University of Marburg, Germany). All experiments were performed according to the German animal protection law.

### *In vivo* administration of IL-2/JES6-1 complex

Wild-type (WT) and *rel^−/−^* mice were treated with three daily injection (days 0,1, 2) of recombinant mouse IL-2 (eBioscience) with anti-IL-2 mAb (clone JES6-1, eBioscience). Recombinant IL-2 was mixed with JES6-1 and incubated for 30 min at 37°C as previously described [23]. Mice were daily i.p. injected with 6 μg IL-2/JES6-1 complex at optimal molar ratio of approximately 2:1 (1 μg/58 pmol of IL-2 and 5μg/33 pmol of JES6-1) and the phenotype of expanded cells was analysed on day 5. For kinetics studies, mLN, spleen and liver cell suspensions were analysed on day 0, 5 and 8.

### Intracellular flow-cytometry

Single cell suspensions were performed from spleens, mLN and iLN by mechanical disruption and passage through pre-separation filter. Colonic lamina propria mononuclear cells (LPMCs) were isolated as previously described [61]. Subsequently, cells were stained with anti-CD4 (eBioscience), fixed and permeabilazed in 3% saponine buffer before intracellular staining. Foxp3- and Helios-expressing cells were routinely detected by using the Foxp3 staining kit (eBioscience), anti-Foxp3 (FJK-16s, eBioscience) and anti-Helios (22F6, eBioscience) Abs. Intracellular detection of IL-17A (eBio17B7) and IFN-γ (XMG1.2) was performed after restimulation of cells with PMA (50 mg/ml)/ionomycin (750 ng/ml) in the presence of brefeldin A (10 mg/ml, all substances were obtained from Sigma-Aldrich) for 4 h. Both Abs were obtained from eBioscience. The stained cells were analyzed by flow cytometry using the FlowJo software (Tree Star).

### Induction of acute colitis

Colitis was induced in sex- and age-matched WT and *rel^−/−^* mice by giving them *3%* (w/v) dextran sodium sulfate (*DSS*, molecular weight 35-50 kDa, MP Biomedicals). DSS was administered orally into the drinking water for 5 days. The body weight was monitored throughout the experiment for 14 days.

### CD4^+^ purification and *in vitro* stimulation

Naïve CD4^+^ T cells were purified from spleens and LN by using CD4^+^ T cell purification kit (Miltenyi Biotec). The cells were activated by plate-bound anti-CD3 (5 mg/ml, clone 145-2C11) and soluble anti-CD28 (1.5 mg/ml, clone 37.51) and differentiated towards Foxp3-expressing Tregs by adding 2 ng/ml TGF-β1 (Peprotech), anti-IL-4 (10% culture supernatant of clone 11B11), anti-IFN-γ (5 μg/ml, clone XMG1.2) and 50 U/ml recombinant hIL-2 (Novartis). After 3 days, the expression of Foxp3 was analyzed by flow cytometry using anti-Foxp3.

### Immunohistochemistry

Immunohistochemistry analysis of Foxp3 expression in the gut of WT and *rel^−/−^* mice was performed as previously described [62]. In brief, 1-2 μm sections of formalin-fixed, paraffin-embedded tissue were cut, deparaffinized, and subjected to a heat induced epitope retrieval step. Slides were rinsed in cool running water, washed in Tris-buffered saline (pH 7.4) before incubation with anti-mouse Foxp3 Ab (clone FJK-16s, eBioscience, dilution 1:50) for 30 minutes. For detection, rabbit anti-rat Ab (Dianova) was used followed by the EnvisionPO kit (DAKO). Peroxidase was developed with a highly sensitive diaminobenzidine chromogenic substrate for 5 minutes. Negative controls were performed by omitting the primary antibodies. Images were acquired using an AxioImager Z1 microscope and processed with AxioVision software (Carl Zeiss MicroImaging).

### Western blot analysis

To study Stat5 phosphorylation, whole-cell lysates were prepared from purified CD4^+^ T cells as described earlier (19). In brief, the Complete^TM^ protease inhibitor cocktail (Roche Applied Science) and phosphatase inhibitors (0.5 mM Na_3_VO_4_ and 50 mM NaF) were added into RIPA lysis buffer. To determine cellular protein content, the Micro BCA Protein Assay Kit (Pierce) was used. For detection of phospho-STAT5, WT and *rel^−/−^* CD4^+^ T cells were isolated from spleen and LN of WT and *rel^−/−^* mice, rested for 3 h, and stimulated *in vitro* for 0–30 min with recombinant hIL-2 (50 U/ml, Novartis). In some experiments, CD4^+^ T cells were cultured under Treg-inducing conditions for 6 h and18 h before the western blot analysis, respectively. The induction of p-STAT5 was examined by using anti-p-STAT5 Ab (Tyr 694, Cell Signaling Techology). Detection of β-Actin (Sigma) and STAT5A (Santa Cruz Biotechnology) served as loading control.

### Statistics

Results are generally expressed as the mean ± SEM. For statistical analysis, data were analyzed using the unpaired two-tailed Student's *t* test. Results were considered statistically significant at P ≤ 0.05. F test was used to determine factorial analysis of variances between DSS-treated and untreated groups. All analyses were performed using GraphPad Prism 5.0 (GraphPad Software).

## SUPPLEMENTARY MATERIALS FIGURES


