# Supplementary material for: Transcription factor c-Rel is indispensable for generation of thymic but not of peripheral Foxp3+ regulatory T cells
Source: Oncotarget. 2017 Apr 13;8(32):52678–89. doi: 10.18632/oncotarget.17079 (PMC5581060; doi:10.18632/oncotarget.17079)
Supplement: Supplementary file 1 [file oncotarget-08-52678-s001.pdf]

Transcription factor c-Rel is indispensable for generation of thymic but not of peripheral Foxp3$^+$ regulatory T cells

SUPPLEMENTARY FIGURES

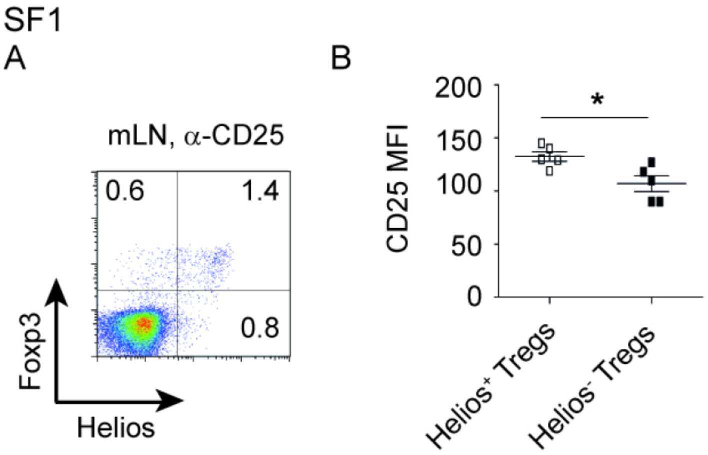

Supplementary Figure 1: (A) The percentage of Tregs in mLN of WT mice following depletion of CD25$^+$ cells. The percentage of Foxp3$^+$CD4$^+$ T cells was analysed by flow cytometry on day 10 after two i.p. injections of 500 µg PC61 mAb (on day 0 and 6, respectively). A dot plot is a representative of two independent experiments (gated on CD4$^+$ cells). **(B)** CD25 MFI values for *ex vivo* Helios$^+$ and Helios$^-$ Tregs derived from mLN of WT mice. Clone PC61.5 was used for FACS analysis. Results are displayed as means ± SEM; *P<0.05.

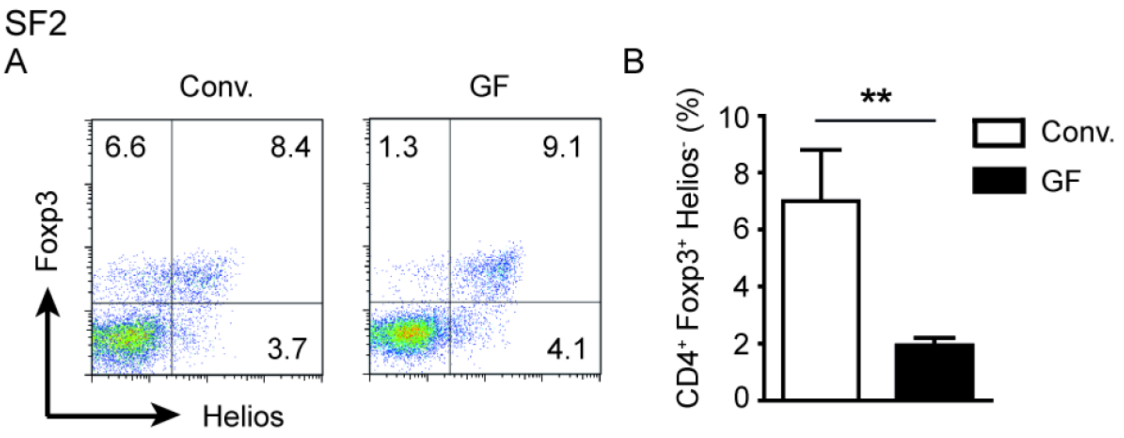

Supplementary Figure 2: (A) and **(B)** Frequencies of Helios^{+}Foxp3^{+} and Helios^{-}Foxp3^{+} Tregs in the colon of conventional (Conv.) and germ-free (GF) mice on C57BL/6 background. Cells are gated on the CD4^{+} gate. A representative of three independent experiments is shown (A). Data in (B) are means ± SEM; $**P<0.01$.

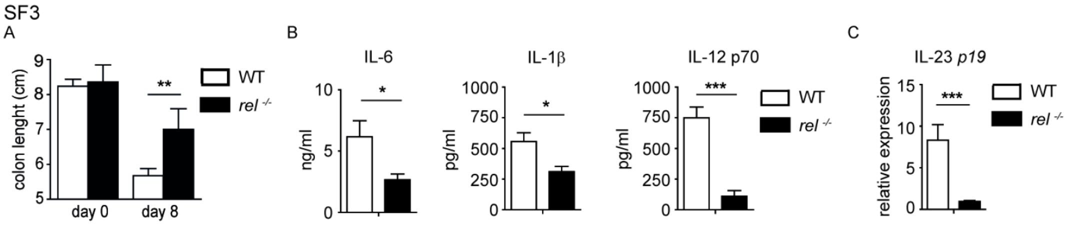

Supplementary Figure 3: (A) Measurement of the colon length in WT and *rel*$^{-/-}$ mice was performed at the indicated time points after the induction of colitis with 3 % DSS (n= 8 mice per group). **(B)** At day 8 after colitis induction, colon explants (n= 8 per group) of DSS-treated *rel*$^{-/-}$ and WT mice were cultured overnight and the concentration of indicated cytokines was measured by ELISA (All ELISA kits were purchased from eBioscience). **(C)** Quantitative RT-PCR analysis of IL-23 *p19* mRNA expression at day 8 after induction of colitis by 3 % DSS (n= 8 per group). The following primers were used for quantitative RT-PCR: IL-23 *p19* primers; forward AGCGGGACATATGAATCTACTAAGAGA; reverse GTCCTAGTAGGGAGGTGTGAAGTTG. For all experiments, the significance was determined by Student`s *t*-test. Data are displayed as means ± SEM. Significance levels: n.s., not significant; * $p<0.05$; ** $p<0.01$; *** $p<0.001$.

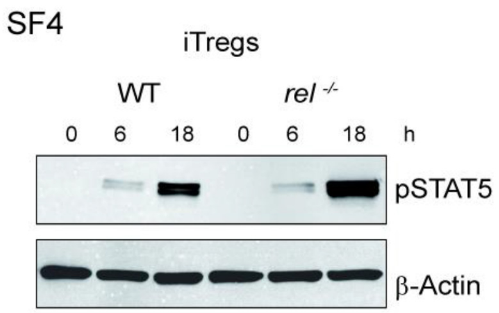

Supplementary Figure 4: Western blot analysis of phospho-Stat5 and β-Actin in WT and *rel*$^{-/-}$ CD4$^+$ T cells isolated from spleen and LN. Total CD4$^+$ T cells were cultured under optimal Treg-inducing conditions with IL-2 (50 U/ml) and TGF-β1 (2 ng/ml). Two similar experiments were performed.
